# Paramedic assessment of pain in the cognitively impaired adult patient

**DOI:** 10.1186/1471-227X-9-20

**Published:** 2009-10-06

**Authors:** Bill Lord

**Affiliations:** 1 Monash University, Department of Community Emergency Health and Paramedic Practice, Building H, McMahons Road, Frankston VIC 3199, Australia

## Abstract

**Background:**

Paramedics are often a first point of contact for people experiencing pain in the community. Wherever possible the patient's self report of pain should be sought to guide the assessment and management of this complaint. Communication difficulty or disability such as cognitive impairment associated with dementia may limit the patient's ability to report their pain experience, and this has the potential to affect the quality of care. The primary objective of this study was to systematically locate evidence relating to the use of pain assessment tools that have been validated for use with cognitively impaired adults and to identify those that have been recommended for use by paramedics.

**Methods:**

A systematic search of health databases for evidence relating to the use of pain assessment tools that have been validated for use with cognitively impaired adults was undertaken using specific search criteria. An extended search included position statements and clinical practice guidelines developed by health agencies to identify evidence-based recommendations regarding pain assessment in older adults.

**Results:**

Two systematic reviews met study inclusion criteria. Weaknesses in tools evaluated by these studies limited their application in assessing pain in the population of interest. Only one tool was designed to assess pain in acute care settings. No tools were located that are designed for paramedic use.

**Conclusion:**

The reviews of pain assessment tools found that the majority were developed to assess chronic pain in aged care, hospital or hospice settings. An analysis of the characteristics of these pain assessment tools identified attributes that may limit their use in paramedic practice. One tool - the Abbey Pain Scale - may have application in paramedic assessment of pain, but clinical evaluation is required to validate this tool in the paramedic practice setting. Further research is recommended to evaluate the Abbey Pain Scale and to evaluate the effectiveness of paramedic pain management practice in older adults to ensure that the care of all patients is unaffected by age or disability.

## Background

Although pain is a commonly encountered complaint in prehospital and emergency medicine settings, evidence of inadequate analgesia has been widely documented. Poor pain management practice has been described in the emergency department (ED)[1], and variations in pain management practice in this setting have been associated with ethnicity[2], gender[3], and extremes of age[4].

Reasons for inadequacies in pain management practice are likely to be multifactorial. Failure to assess for the presence and severity of pain may be one factor, as efforts to make pain measurement mandatory in the ED have been shown to improve the frequency of analgesic administration[5]. The importance of early and systematic assessment of pain is exemplified by recommendations to include pain as the "5^th ^vital sign"[6], reinforcing the need to seek and record evidence of pain in every patient encounter. However, even when pain assessment is encouraged or required, patients may be unable to communicate their experience to carers, or be reluctant to report pain due to concerns about treatment side effects or the possibility that they will be viewed as a complaining or difficult patient, a belief that has been documented in settings that include oncology [7] and aged care[8,9].

Paramedics have an important role in the assessment and management of pain, and are often a first point of contact for people experiencing pain in the community. Effective management of pain in this context is made possible by evidence-based clinical practice guidelines that enable paramedics to relieve pain by pharmacological and non-pharmacological means. However, effective management of pain depends on the paramedic's ability to gather relevant clinical information to reveal the presence, nature and severity of the patient's pain. As pain is a personal experience with external manifestations that are associated with significant interpersonal variations of expression[10] that limit generalisations regarding standards of pain behaviour, wherever possible the patient's self report of pain should be sought to guide the clinician's assessment and management of this complaint[11].

Pain severity is one component of a complex and highly personal experience that involves sensory-discriminative, motivational-affective and cognitive-evaluative dimensions[12]. Assessment of pain severity is specifically sought to guide paramedics' pain management decisions, which may include strategies designed to mitigate the cause of the pain and to provide relief from pain that includes efforts to manage the environmental, social and psychological mediators of the perception and expression of pain[10]. In addition, the assessment and evaluation of the patient's pain experience will influence pharmacological interventions aimed at providing relief from pain.

Tools used to elicit a patient report of severity include the Verbal Descriptor Scale (VDS), which requires the patient to rate their pain using adjectives such as "none," "slight," "moderate," "severe," or "agonizing," and the Verbal Numeric Rating Scale (VNRS), where the patient assigns a number from 0-10 to quantify their pain, with 0 representing no pain and 10 representing the worst pain imaginable. Both types of scale are recommended for use by paramedics[13]. The Visual Analogue Scale (VAS) has also been used to measure pain severity in adults in the prehospital setting[14,15].(In Australia the Victorian Ambulance Service recommends the use of the VNRS for the assessment of pain in adults[16], and in the United Kingdom, the clinical practice guidelines developed by the Joint Royal Colleges Ambulance Liaison Committee also recommends the use of the VNRS for scoring pain severity in adult patients[17].

While these scales have been shown to be valid methods of documenting pain severity and changes in severity, their effectiveness depends on the patient's ability to understand instructions in their use in order to quantify their pain. In addition, self-report of pain severity requires the use of higher cognitive functions and the ability to use abstract reasoning to associate numbers or a list of adjectives with the severity of pain that an individual may be experiencing. While many patients can use these scales to indicate the severity of their pain, in others the ability to communicate their pain experience may be impaired by language difficulties, developmental barriers (developmental disability and pre-verbal children), physiological barriers (for example coma), or cognitive barriers that include diseases such as dementia. These problems can pose special challenges for health professionals seeking to establish the nature and severity of the patient's distress, and this has the potential to result in suboptimal care.

Evidence to support this assertion may be found in a recent study involving a large number of nursing home residents (n = 551), which revealed that the incidence of nursing staff records of pain in residents declined as cognitive disability increased[18]. While 34% of patients with no cognitive disability reported pain during the study period, pain prevalence rates of 31%, 24%, and 10% were associated with residents with mild, moderate, and severe cognitive impairment. Furthermore, as cognitive disability increased the administration of analgesics decreased, despite there being no statistical difference in the prevalence of painful pathologies between cognitively impaired and cognitively normal residents. This suggests that the higher the level of cognitive impairment the more difficult it is to record or report pain.

The results may also illustrate a lack of willingness to seek evidence of pain in individuals where communication difficulties complicate the assessment process. A similar result has been observed in an earlier study that found a decrease in the prescription and administration of analgesics in cognitively impaired nursing home residents despite similar proportions of painful pathologies in the impaired and non-impaired cohorts[19].

Dementia is a major cause of cognitive impairment in adults. Many developed countries are experiencing a rapidly aging population, and as dementia is an age-related disease, the prevalence of dementia in many countries is predicted to increase. For those living in Australia who are aged more than 65, the likelihood of having dementia doubles every five years, so that by age 85 it is estimated that 24% of people are affected[20]. The prevalence in this country is estimated to increase from approximately 175,000 in 2003 to approximately 465,000 by 2030[21]. Although this disease may impair an individual's ability to report pain, the ability to feel pain may remain unimpaired[22,23].

The increasing prevalence of this disease means that more people may be at risk of living with pain that cannot be adequately reported to others, making the need to establish a valid and reliable means of identifying pain in this population a priority, as failure to identify pain and subsequently implement strategies to relieve a patient's pain may be considered a form of medical error and a denial of a basic human right[24,25].... As tools currently used by paramedics to assess pain may be unreliable in the presence of cognitive impairment this paper aims to identify tools that may assist paramedics to assess these challenging cohorts of patients in order to ensure that their pain is recognised, thereby enabling interventions aimed at relieving their pain. The primary objective of this review was to systematically locate evidence relating to the use of pain assessment tools that have been validated for use with cognitively impaired adults and to identify those that have been recommended for use by paramedics. A secondary objective was to make recommendations regarding the paramedic assessment of pain in cognitively impaired individuals if no existing recommendations could be found. The focus will be the assessment of pain in people with cognitive impairment due to dementia, as this represents the major cause of cognitive impairment in older adults.

## Method

In order to locate evidence relating to the research questions the following databases were searched over the period January 1985 through June 2008: Medline, Cumulative Index to Nursing & Allied Health (CINAHL), Biological Abstracts, and Psycinfo. The search included key words and/or medical subject headings (pain measurement OR pain assessment) AND (dementia OR cognition disorders/cognitive impairment OR nonverbal communication).

An extended search was subsequently conducted of the electronic database of the National Guideline Clearinghouse to identify guidelines on pain assessment in older adults, particularly those recommended for the assessment of the nonverbal older adult or those with dementia. In addition, position statements and clinical practice guidelines were sought through searches of relevant Internet sources such as the International Association for the Study of Pain, the Australian Pain Society, and the National Health and Medical Research Council.

Due to the large number of research reports that were located using the initial search strategy it was decided to restrict the search to reports that met the following criteria:

### Type of studies

Systematic reviews.

### Participants

Cognitively impaired adult patients suspected of having acute or chronic pain in a clinical setting.

### Interventions

Assessment of pain using a previously developed tool that claimed to assess one or more dimensions of the patient's pain experience, including pain severity.

### Outcomes

Measures of validity, reliability and practicality of the pain assessment tools.

## Results

The search strategy returned 575 results:

1 pain measurement.mp. or Pain Measurement/(48729)

2 pain assessment.mp. (11225)

3 Dementia/or dementia.mp. (111623)

4 Cognition Disorders/or cognitive disorders.mp. (46458)

5 cognitive impairment.mp. (34158)

6 nonverbal communication.mp. or Nonverbal Communication/(5621)

7 1 or 2 (53402)

8 limit 7 to (english language and humans and yr="1985 - 2008")(43422)

9 3 or 4 or 5 or 6 (165940)

10 limit 9 to (english language and humans and yr="1985 - 2008") (129860)

11 8 and 10 (857)

12 remove duplicates from 11 (575)

When the search result was limited using keywords "paramedic" OR "emergency medical technician" OR "ambulance/s" OR "prehospital" OR "emergency medical services", there were no (0) results.

The full-text versions of studies that matched the initial inclusion criteria were reviewed. This strategy identified two reports that met the selection criteria:

• Herr K, Bjoro K, Decker S:**Tools for assessment of pain in nonverbal older adults with dementia: a state-of-the-science review**. J Pain Symptom Manage 2006, **31**:170-92.

• Zwakhalen SM, Hamers JP, Abu-Saad HH, Berger MP:**Pain in elderly people with severe dementia: a systematic review of behavioural pain assessment tools**. BMC Geriatr 2006, **6**.

### Analysis and evaluation of the systematic reviews

Herr and colleagues used the following selection criteria for their systematic review:

1. Studies based on behavioural indicators of pain;

2. Developed for assessment of pain in nonverbal older adults with severe dementia or evaluated for use with nonverbal older adults;

3. Available in English; and

4. At least one published research report of psychometric evaluation available in English[26].

These criteria identified 10 behaviourally-based pain assessment tools for use with older adults with dementia. The tools were evaluated in each of the areas of "conceptualization, subjects, administration, reliability, and validity." The authors independently critiqued each tool and applied a score from 0-3 for each of the five evaluation categories, with a score of 3 indicating strong evidence for each construct to 0 for no evidence. Studies that described the implementation and evaluation of the 10 tools were analysed and the strengths and limitations noted to arrive at a total score for each tool. This process revealed that only one tool has been tested with older adults in acute care settings (the Abbey Pain Scale)[27].

The authors concluded that, while some tools are potentially useful, weaknesses in the tools evaluated mean that there is currently "no standardized tool based on nonverbal behavioural pain indicators in English that may be recommended for broad adoption in clinical practice"[26]. One reason given for this conclusion was the acknowledgment that the ability to recognise pain and rate pain severity on the basis of behavioural cues is limited by significant inter-patient variability in pain-related behaviours that may also be affected by co morbid conditions such as stroke and psychiatric illness.

The study by Zwakhalen et al used a more comprehensive scoring method that, in addition to the categories evaluated by Herr et al, included an evaluation of study size and homogeneity of studies. The expanded range of scores for each of the constructs being evaluated produced a total possible score of 20. The authors evaluated seven of the tools reported by Herr and colleagues, and evaluated an additional five tools that were not included in the former study, before recommending the Pain Assessment Checklist for Seniors with Limited Ability to Communicate (PACSLAC)[28] and DOLOPLUS-2[29]. scales as the most appropriate scales currently available.

The difference in results between these two studies reflects differences in evaluation methodology. For example, the highest rating tool in the Herr et al study was the DS-DAT, but this tool was excluded from the study by Zwakhalen and colleagues as this tool attempted to rate discomfort rather than pain, and was therefore conceptually different than other tools designed to evaluate pain in this population. Differences in the study results may also reflect a lack of consensus on how to validate tools for observational assessment of pain behaviours.

In addition to the literature search already described an Internet search for paramedic clinical practice guidelines or documents that were not cited in the search databases was undertaken, but this failed to identify any evidence of tools for the assessment of pain in adults with cognitive impairment that are recommended for use by paramedics in community emergency care settings.

Reference to cognitive impairment and the consequent impact this condition has on the paramedic's ability to assess pain is rarely mentioned in the paramedic literature. Although no specific recommendations were found regarding the paramedic assessment of pain in cognitively impaired individuals, there was some evidence of general advice regarding the need to assess cues such as behaviour in the absence of a self-report. The clinical practice guidelines that inform paramedic practice in the United Kingdom advise that the use of pain assessment tools such as the VNRS in the assessment of patients with cognitive impairment may be difficult, and recommend that "in these circumstances behavioural cues will be more important in assessing pain"[30]. However, no further guidance is provided regarding the types of behavioural cues that are strongly correlated with pain and pain severity.

## Discussion

The reviews of pain assessment tools for the cognitively impaired that were included in the cited systematic reviews show that the majority were developed to assess chronic pain in aged care, hospital or hospice settings. An analysis of the characteristics of these pain assessment tools identified attributes that may limit their use in paramedic practice. These include assessment that is possibly too comprehensive and time consuming for paramedics to perform. For example, several tools included in the systematic reviews are recommended for use in aged care institutions and involve complex scoring that requires repeated observation of patient behaviours over time by trained observers. Some, such as the NOPAIN tool[31], are designed to be used while observing the patient undertaking daily tasks such a dressing and bathing, which restricts its use by paramedics.

The DOLOPLUS-2[29] scale requires observation of patient behaviour over time in several different situations including social interactions and sleep. Its use is limited in the acute setting as the patient's normal behaviour must be well known to the carers who complete the assessment. A recent review of this tool has questioned its validity and has identified the considerable administrative demands required to assess pain behaviours[32].

Assessment of pain using PACSLAC[28] involves observation of 60 items that include behaviour during movement, eating and sleeping as well as mood and changes in social interactions. This tool also requires observation of the patient over time to enable observation of often subtle changes in behaviour. As such this tool is likely to be impractical for paramedic use.

One behaviourally-based pain assessment tool that is currently used by paramedics in the Australian state of Victoria is the Face, Legs, Activity, Cry and Consolability (FLACC) tool, which is used to assess pain in nonverbal children[33]. Although there is some evidence of the use of this tool for assessment of pain in cognitively impaired older adults[26], this tool may not be appropriate for the assessment of pain in this population. The FLACC scale was developed to guide the assessment of pain in infants and pre-verbal children, and the pain-related behaviours that form the basis of this tool were identified from studies of children undergoing painful procedures such as circumcision. Some behaviour addressed by this scale such as leg kicking and a quivering chin does not appear to be relevant when assessing adults. The review of adult pain assessment tools undertaken by Herr and colleagues found that the FLACC has low levels of validity and reliability and as such was not recommended for use in this population[26].

**Figure 1:**
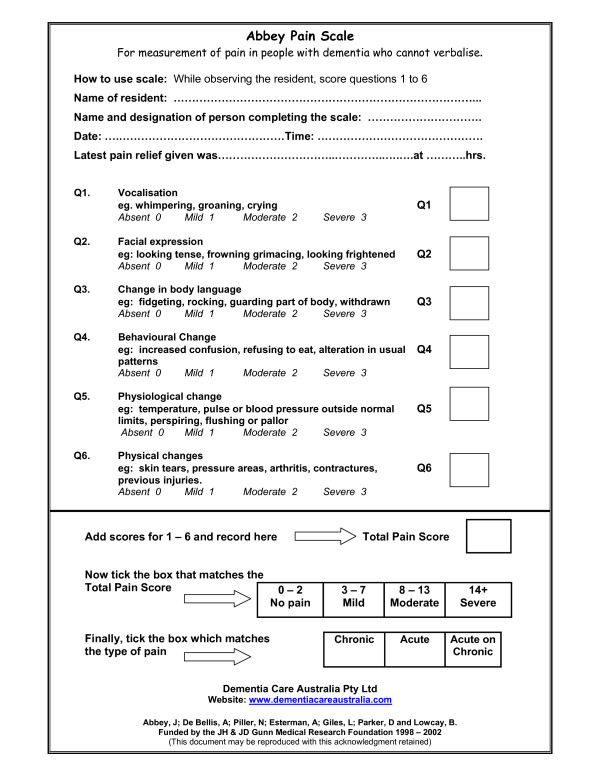
**Abbey Pain Scale**. From: Abbey J et al. The Abbey pain scale: a 1-minute numerical indicator for people with end-stage dementia [27].

Any tool used by paramedics must be reliable, valid and practical, with the latter influenced by operational requirements to minimise time spent on scene. As such, tools that assess multiple dimensions of pain that require observation of behaviour over time during different activities may have less utility than a tool that identifies the presence of pain and attempts to evaluate the severity in a way that parallels tools that are already familiar to paramedics for use in patients without cognitive impairment. In a report published by the Australian Pain Society[34] that describes the use of best available evidence and the results of a clinical trial of pain assessment tools to inform pain management practice in aged care facilities, the Abbey pain scale (Figure 1) was recommended as the most appropriate means of assessing pain in residents with severe cognitive impairment. This one-dimensional scale is designed to rate pain severity. Although this tool attempts to address acute, chronic and acute-on-chronic pain using six behaviour categories that include physiological and physical changes, vocalisation, facial expressions, and changes in body language and behaviour, some cues may be non-specific. This is particularly apparent in the facial cue category, where cues such as frowning may not have a strong correlation with pain[35]. The tool takes between two to six minutes to complete[36], and as such this tool may be practical for use in the paramedic practice setting.

Following a recent review of available evidence the Abbey Pain Scale was recommended by the Royal College of Physicians in the UK for assessment of pain in patients with severe cognitive impairment[37]. The authors of this report recognised that limited clinical data was available to support this decision, but made this recommendation on the basis on ease of use while adding the caveat that no single method of pain assessment could be recommended for this cohort.

At this time no pain assessment tools for use in the setting of cognitive impairment have been validated for use by paramedics. Until studies of the paramedic use of tools such as the Abbey Pain Scale are undertaken, general recommendations can be made to aid the assessment of pain in patients with cognitive impairment. The following clinical practice recommendations are adapted from the American Society of Pain Management Nursing Position Statement on Pain Assessment in the Nonverbal Individual[38]. Given the evidence that establishes a link between cognitive impairment and reduced pain management interventions, paramedics need to be proactive in seeking evidence of pain in this vulnerable population. Strategies that may be employed to improve the identification of pain in cognitively impaired adults include assessment of injuries associated with pain, interpretation of behaviour, surrogate estimation of pain by carers or close family members, use of a pain assessment tool, and observation of clinical response to analgesics or other non-pharmacological interventions designed to relieve pain. However, no single assessment strategy is sufficient by itself[38].

### 1. Identify possible causes of pain

The likelihood of pain may be inferred by the presence of injury or disease that is normally associated with pain. Where the patient has an obvious recent fracture or dislocation, extensive soft tissue injury due to a fall or from burns and scalds, the patient is likely to be experiencing pain even though they may be unable to clearly communicate this. Assessment of pain may be aided by evidence of a pattern of injury such as the limb shortening and external rotation frequently associated with fractures to the neck of the femur. There is no strong evidence that patients with dementia suffer less pain, with some evidence suggesting that patients with dementia suffer more pain than those without cognitive impairment[39]. However, paramedics may not consider the need for analgesia if they believe that cognitive impairment is associated with reduced pain perception.

Where the patient's behaviour suggests the presence of pain but the cause is less obvious, such as pain arising from ischaemia of visceral organs, the confirmation of pain is more difficult. The assessment may also be complicated by chronic pain from conditions such as arthritis and osteoporosis, or from cancer or recent surgical procedures. However, pain may have no identifiable pathological basis, and confirmation of an injury or disease process to account for the pain is not needed. Withholding analgesia in the absence of an obvious source is inappropriate where other clinical cues suggest that the patient is experiencing pain.

### 2. Observe patient behaviour

Assessment of pain in the cognitively impaired adult may require the establishment of individual benchmarks for behaviour. This is done by asking carers, relatives or close friends to describe normal behaviour and any recent changes in the patient's behaviour. Where the patient is a resident of an aged care facility the nursing staff should be questioned regarding the use of pain assessment tools, and if used, whether an attempt has been made to assess the patient to identify evidence of pain.

Some behaviours have been shown to be associated with pain, and these include facial expressions, vocalising, certain body movements, and changes in interpersonal interactions or in activity or daily routines[40]. While pain assessment tools should attempt to address each of these behaviours, the assessment of some requires evidence of prior behavioural norms and observation of behavioural changes over time. For paramedics called to see patients with the potential presence of pain this information may unavailable, and observation over time impractical given the operational pressures to minimise scene and transport times. However, facial expressions may be an important indicator of pain, with evidence that prototypical facial expressions of pain are reliably identified by observers of another individual's pain-related expressions, and that observers are able to discriminate between facial expressions associated with pain and those associated with other emotions such as fear[35]. In an experimental pain setting the facial responses of patients with dementia and those in the healthy control group were closely related to the intensity of the stimulation, leading to a conclusion that facial expression may be an important pain assessment tool in patients with impaired cognition or inability to self-report their pain experience[41]. Facial changes associated with pain have been shown to be consistent across the lifespan [42], and as the identification of facial cues does not require the establishment of base rate data or trends in behaviour this may be an important cue that can be assessed by paramedics in order to identify the presence of pain. In addition, this does not demand assessment over time as is required by some other behavioural cues.

### 3. Seek information from others

Information should be sought from the patient's family, close friends or carers regarding changes in behaviour that may be associated with the presence of pain. People who know the patient well are likely to be able to report subtle changes in the patient's behaviour or daily activities that may suggest pain. This use of surrogate reporting of pain has some advantages over a naive assessment of pain. However, evidence shows a tendency for doctors[43] and allied health professionals to underestimate the severity of the patient's pain experience[44,45]... This phenomenon has also been observed in the prehospital setting[46]. As such the use of surrogate measures of pain should be supported by other clinical evidence wherever possible.

### 4. Use a pain assessment tool

Although the patient's ability to use pain assessment tools such as the VNRS and VNRS depends on the extent of cognitive impairment, patients should still be asked to provide an assessment of their pain using these tools as there is evidence that they may be successfully used in patients with mild to moderate cognitive impairment[47]. Other barriers to communication not related to dementia - such as hearing loss - should be considered and aids used to ensure that the communication problem is not related to another disability before considering the use of a pain assessment tool designed for patients with cognitive impairment.

### 5. Consider an analgesic trial

If all the available evidence suggests that the patient is experiencing pain and other interventions have failed to relieve the pain it may be reasonable to administer an analgesic to observe the response this has on pain-related behaviours. Patients with moderate to severe cognitive impairment due to dementia may have difficulty understanding instructions regarding the self-administration of inhalational analgesics such as nitrous oxide or methoxyflurane, and as such small aliquots of a parenteral analgesic may be required. While it is important to be guided by principles of beneficence and to adopt a humanitarian approach to relieving pain and suffering, of equal importance is the need to minimise harm arising from unnecessary administration of analgesics in response to a false positive arising from an assessment of the presence of pain. Unlike other forms of diagnostic tests there is no gold standard tool for confirming the variable and very personal experience of pain.

## Conclusion

Paramedics have the tools to relieve pain in the form of effective pharmacological - opioid and non-opioid - and non-pharmacological adjuncts. However, equitable and effective management of pain relies on the self-report of this symptom. In patients whose self-report is limited by cognitive disability paramedics may need to use other methods of seeking evidence of pain. A patient who cannot clearly articulate their pain experience is just as deserved of relief from pain as those who are not burdened with disability. While some pain assessment tools have been recommended for use in patients with cognitive impairment there is currently lack of consensus on the most appropriate tool to use. As such, research is recommended that aims to test the utility, validity and reliability of the Abbey Pain Scale in identifying pain in this at-risk population in the prehospital setting. Further research should also evaluate the effectiveness of paramedic pain management practice in older adults to ensure that the care of all patients is unaffected by age or disability.

## Competing interests

The author declares that he has no competing interests.

## Pre-publication history

The pre-publication history for this paper can be accessed here:


